# Exploratory Data Mining Techniques (Decision Tree Models) for Examining the Impact of Internet-Based Cognitive Behavioral Therapy for Tinnitus: Machine Learning Approach

**DOI:** 10.2196/28999

**Published:** 2021-11-02

**Authors:** Hansapani Rodrigo, Eldré W Beukes, Gerhard Andersson, Vinaya Manchaiah

**Affiliations:** 1 School of Mathematical and Statistical Sciences University of Texas Rio Grande Valley Edinburgh, TX United States; 2 Virtual Hearing Lab Beaumont, TX United States; 3 Department of Speech and Hearing Sciences Lamar University Beaumont, TX United States; 4 Department of Vision and Hearing Sciences School of Psychology and Sport Science Anglia Ruskin University Cambridge United Kingdom; 5 Department of Behavioral Sciences and Learning Department of Biomedical and Clinical Sciences Linköping University Linköping Sweden; 6 Department of Clinical Neuroscience Division of Psychiatry Karolinska Institute Stockholm Sweden; 7 Department of Speech and Hearing School of Allied Health Sciences Manipal Academy of Higher Education Karnataka India; 8 Department of Speech-Language Pathology and Audiology University of Pretoria Gauteng South Africa

**Keywords:** tinnitus, internet interventions, digital therapeutics, cognitive behavioral therapy, artificial intelligence, machine learning, data mining, decision tree, random forest

## Abstract

**Background:**

There is huge variability in the way that individuals with tinnitus respond to interventions. These experiential variations, together with a range of associated etiologies, contribute to tinnitus being a highly heterogeneous condition. Despite this heterogeneity, a “one size fits all” approach is taken when making management recommendations. Although there are various management approaches, not all are equally effective. Psychological approaches such as cognitive behavioral therapy have the most evidence base. Managing tinnitus is challenging due to the significant variations in tinnitus experiences and treatment successes. Tailored interventions based on individual tinnitus profiles may improve outcomes. Predictive models of treatment success are, however, lacking.

**Objective:**

This study aimed to use exploratory data mining techniques (ie, decision tree models) to identify the variables associated with the treatment success of internet-based cognitive behavioral therapy (ICBT) for tinnitus.

**Methods:**

Individuals (N=228) who underwent ICBT in 3 separate clinical trials were included in this analysis. The primary outcome variable was a reduction of 13 points in tinnitus severity, which was measured by using the Tinnitus Functional Index following the intervention. The predictor variables included demographic characteristics, tinnitus and hearing-related variables, and clinical factors (ie, anxiety, depression, insomnia, hyperacusis, hearing disability, cognitive function, and life satisfaction). Analyses were undertaken by using various exploratory machine learning algorithms to identify the most influencing variables. In total, 6 decision tree models were implemented, namely the classification and regression tree (CART), C5.0, GB, XGBoost, AdaBoost algorithm and random forest models. The Shapley additive explanations framework was applied to the two optimal decision tree models to determine relative predictor importance.

**Results:**

Among the six decision tree models, the CART (accuracy: mean 70.7%, SD 2.4%; sensitivity: mean 74%, SD 5.5%; specificity: mean 64%, SD 3.7%; area under the receiver operating characteristic curve [AUC]: mean 0.69, SD 0.001) and gradient boosting (accuracy: mean 71.8%, SD 1.5%; sensitivity: mean 78.3%, SD 2.8%; specificity: 58.7%, SD 4.2%; AUC: mean 0.68, SD 0.02) models were found to be the best predictive models. Although the other models had acceptable accuracy (range 56.3%-66.7%) and sensitivity (range 68.6%-77.9%), they all had relatively weak specificity (range 31.1%-50%) and AUCs (range 0.52-0.62). A higher education level was the most influencing factor for ICBT outcomes. The CART decision tree model identified 3 participant groups who had at least an 85% success probability following the undertaking of ICBT.

**Conclusions:**

Decision tree models, especially the CART and gradient boosting models, appeared to be promising in predicting ICBT outcomes. Their predictive power may be improved by using larger sample sizes and including a wider range of predictive factors in future studies.

## Introduction

### Background

Tinnitus is the perception of a sound in the ears or head in the absence of a corresponding external sound source. It is very prevalent; it has been estimated that 10% to 15% of the adult population experiences tinnitus [1]. Various conditions are associated with developing tinnitus, such as ear disorders [2], exposure to loud noise, the presence of hearing loss, and increasing age [3]. Tinnitus experiences are highly heterogeneous in terms of how it manifests (eg, the types of sounds experienced, how individuals react to their sounds, the associated comorbidities, etc) and how individuals with tinnitus respond to treatment [4]. Although a majority of those with tinnitus are not bothered by their tinnitus, a significant number of people with tinnitus experience distressing tinnitus that affects their quality of life [5]. Nevertheless, tinnitus can affect people in different ways; the most common complaints include annoyance, irritability, fatigue, stress, sleep problems, and trouble concentrating [6]. Moreover, distressing tinnitus is often associated with an increased risk of anxiety and depression [7,8]. Various management strategies are used to help persons with tinnitus, including sound therapy (eg, hearing aids and masking); informational counseling for aiding people’s understanding of tinnitus; and psychological approaches that address unhelpful thought patterns and reactions to tinnitus, such as cognitive behavioral therapy (CBT). Of these, CBT has the highest level of research evidence on reducing tinnitus distress [9,10].

Although the use of CBT is recommended in many tinnitus practice guidelines [11], it is seldomly provided. This is partly due to a lack of trained professionals who can offer CBT for tinnitus in an in-person format. To overcome this barrier, internet-based CBT (ICBT) was developed in the late 1990s [12]. In ICBT, the treatment strategies are offered to individuals with tinnitus as self-help materials that are provided over the internet together with professional guidance [13]. The feasibility and efficacy of such an approach have been demonstrated among several populations in Sweden, Germany, Australia, the United Kingdom [14], and, more recently, the United States [15,16]. In general, studies have shown that nearly 50% to 60% of those who undergo ICBT will experience a clinically significant reduction in tinnitus distress [17,18]. To date, no strong predictors of ICBT outcomes have been identified to indicate who is likely to benefit from ICBT interventions. The predictors of outcomes that were identified when examining the long-term (1 year) outcomes of ICBT in the United Kingdom were higher baseline tinnitus severity, more engagement with the ICBT program (ie, more modules opened), and higher self-reported satisfaction with the intervention [18]. To further explore predictors of outcomes, various univariate and multivariate (ie, logistic and linear) regression models were applied to a combined data set of multiple ICBT studies [19]. These linear and logistic regression models identified education level (linear regression: *P*=.01; logistic regression: *P*<.001) and baseline tinnitus severity (linear regression: *P*<.001; logistic regression: *P*<.001) to be significant predictor variables that contribute to reductions in tinnitus severity following an ICBT intervention. As per the linear regression model, participants who had received disability allowance showed a 25.30-point less (95% CI −46.35 to −4.24) Tinnitus Functional Index (TFI) score reduction when compared to those who did not have to work less due to tinnitus after adjusting for baseline tinnitus severity and participants’ education levels.Although many other predictors, including age and tinnitus duration [19], were not identified to be significant under these linear models, these variables might have a nonlinear association with the response.

In the last 2 decades, various artificial intelligence and machine learning techniques have been developed and applied to hearing health data. Such approaches have mainly been used for disease profiling, although some studies have focused on the prediction of treatment outcomes [20-24]. It is noteworthy that the intervention trials in audiology and tinnitus research usually involve a few hundred participants and the collection of generally extensive data regarding demographic characteristics and clinical variables. Such a data set with many predictor variables may be best handled by exploratory data mining techniques, such as the use of tree-based models (eg, the random forest [RF] model). Such models tend to perform well even in the presence of multicollinearity among a large number of predictor variables, as these models decorrelate the variables [25]. For example, a recent study that examined various machine learning algorithms for predicting CBT treatment outcomes in the tinnitus population suggested that gradient boosted trees (area under the receiver operating characteristic curve [AUC]=0.89) have the best predictive power [21]. This study found that subjectively perceived tinnitus-related impairment, depression, sleep problems, physical health–related impairments in quality of life, the time spent on completing questionnaires, and educational level highly contributed to the model’s predictions. However, no previous studies have examined the application of artificial intelligence and machine learning techniques to ICBT outcomes in tinnitus research.

### Objectives

To further explore outcome predictors for ICBT, this study aimed to examine the applications of various exploratory data mining techniques based on decision tree models. In particular, we wanted to (1) investigate which types of decision tree models were the most applicable to ICBT outcome prediction (ie, models with the best accuracy and predictive power) and (2) identify the most relevant predictive factors of ICBT outcomes by using the most appropriate decision tree models.

## Methods

### Study Design and Ethical Considerations

We included 228 participants who previously underwent ICBT for tinnitus and whose data were collected as a part of 3 separate ICBT trials [17,18,26] that were conducted from 2016 to 2018. This study was a secondary analysis of these ICBT intervention studies. Ethical clearance was obtained from the Faculty of Science and Technology Research Ethics Panel of Anglia Ruskin University (reference numbers: FST/FREP/14/478 and FST/FREP/14/478) and the East of England–Cambridge South Research Ethics Committee (reference number: 16/EE/0148) and Health Research Authority (Integrated Research Application System project ID: 195565).

### Participant Characteristics

A heterogenous sample of individuals with tinnitus was obtained; thus, the sample represented typical tinnitus populations, as seen in Multimedia Appendix 1. The average age was 55.14 years (SD 12.92 years), and 98 out of the 228 (43%) participants were females. The majority of participants (154/228, 67.5%) had long-standing tinnitus with a mean duration of 17.68 years (SD 19.42 years). Of the 228 participants, 59 (25.9%) had completed high school education, 61 (26.8%) had an undergraduate degree, and only 30 (13.2%) had a postgraduate degree. Approximately 48% (109/228, 47.8%) of the participants experienced tinnitus in both ears, 26.8% (61/228) of them experienced tinnitus in 1 ear, and the others reported experiencing tinnitus in their head or in other locations. The majority (159/228, 69.7%) of participants did not wear hearing aids, and 25.4% (58/228) of them had sought tinnitus treatment previously.

### Intervention

The study participants completed an 8-week ICBT intervention that was presented in a self-help format [13,27]. The intervention was administered by using a secure e-platform [28,29]. During this 8-week period, participants were presented with 2 to 3 learning modules that contained various elements of CBT that were specifically adapted for tinnitus, including applied relaxation, cognitive restructuring, and imagery. The digital materials were presented by using text, images, and videos. In addition, various exercises were presented in these learning modules to improve engagement.

### Data Collection

The baseline data collection included an extensive questionnaire that focused on demographics and tinnitus-related and treatment-related information. Outcome data were gathered by using standardized primary and secondary self-reported questionnaires, which were administered before (baseline), during (weekly), and after the intervention. The primary outcome was a change in tinnitus severity, as measured by the TFI [30]. The secondary outcome measures included the Insomnia Severity Index [31] (a measure of insomnia), the Generalized Anxiety Disorder-7 [32] (a measure of anxiety); the Patient Health Questionnaire-9 [33] (a measure of depressive symptoms); the Hearing Handicap Inventory for Adults Screening version [34] (a measure of self-reported hearing disability); the Hyperacusis Questionnaire [35], which was used to assess the presence hyperacusis (ie, reduced tolerance to everyday sounds); the Cognitive Failures Questionnaire [36], which was used to assess cognitive functions; and the Satisfaction with Life Scales [37], which were used to assess global life satisfaction.

### Data Analyses

#### Variables

The primary outcome variable (the dependent variable) in this study was a change in tinnitus severity. A 13-point reduction in TFI scores following the ICBT intervention was regarded as a clinically significant (successful) treatment outcome [30]. Significant differences in scores were assessed by using paired sample *t* tests. All tests were two-tailed, and significance was set to *P*=.05. There were 33 predictor variables selected, as outlined in Multimedia Appendix 2. These included the following:

- 7 demographic variables (ie, age, gender, education level, employment type, noise exposure, the presence of psychological conditions, and tinnitus that affects the ability to work)
- 15 tinnitus and hearing-related variables (ie, baseline tinnitus severity, tinnitus duration, how often tinnitus is heard, tinnitus location, 9 different types of tinnitus, tinnitus in which multiple tones are heard, and the presence of hearing loss)
- 4 treatment-related variables (ie, past treatment sought, tinnitus maskability, hearing aid use, and medication use)
- 7 clinical factors (ie, anxiety, depression, insomnia, hyperacusis, hearing disability, cognitive functions, and life satisfaction).

#### Decision Tree Models (Classifiers)

The data analysis focused on decision tree–based models, as they play an essential role in exploratory data mining and facilitate human decision-making by providing decision rules [38]. Despite their simplicity, decision trees usually exhibit high variance in their predictions and are not consistently robust. Given these issues, their powerful counterparts, such as the RF [39], gradient boosting (GB) [40], and extreme GB (XGBoost) [41], models were selected. For comparison, 6 decision tree models were used, namely the classification and regression tree (CART) [42], C5.0 [43], GB, XGBoost, AdaBoost algorithm [44], and RF models. As the CART, C5.0, and RF decision tree models involve stratifying or segmenting the predictor space into a number of nonoverlapping regions [38], recursive binary splitting for classification via the Gini index was performed [45]. Many of these decision tree types have been applied to audiological data, and they were found to provide good results in previous studies [20-24].

#### Data Analysis Steps

##### Summary of Data Analysis Steps

The analyses were performed in 4 stages. First, the data were split into training and testing data, and the classifier models were trained on a data set before testing. Second, the six classifiers were applied to the test data to identify the most suitable models based on their performance evaluation. Third, the two best models were used to determine the predictors of ICBT outcomes. Fourth, the optimal CART decision tree model was used to identify the participants who were more (or less) likely to benefit from ICBT treatment. The steps are described in more detail below.

##### Step 1: Classifier Training

Prior to applying the decision tree classifiers, the entire data set was divided into the training (183/228, 80.3%) and testing (45/228, 19.7%) data sets. The training data set was used to develop the corresponding data mining model, while the testing data set was used to evaluate the model predictions. As the training data set was relatively small (n=183), a repeated 3-fold cross-validation was performed. This was not done for the CART model, for which the full training data set was used for model training. With this approach, each fold was given a chance to act as their own validation set to minimize the propensity of model overfitting. In total, 10 different models were created with several random initializations for each data mining method. Hyperparameter tuning for each of these decision models was performed, as required. For instance, when training the RF models, we explored a range of different numbers of predictors for the splitting at each tree node and their impact on the models’ performance.

##### Step 2: Classifier Performance Evaluation

The trained models were evaluated (ie, by using the testing data set) in terms of their mean predictive accuracy, sensitivity (true positive rate), specificity (true negative rate), and AUCs. These were presented as means and SDs and based on the 10 replicated models for each data mining technique. The AUC is used as a measurement of model discrimination power. The optimal decision tree models were selected based on having the highest AUC value. In general, models with an AUC of 0.5 have no discriminatory power, models with an AUC of 0.7 to 0.8 are considered acceptable, models with an AUC of 0.8 to 0.9 are considered excellent, and models with an AUC of >0.9 are deemed to have outstanding discriminatory power (ie, the ability to identify patients with and without a disease or condition based on a new set of data).

##### Step 3: Predictors of ICBT Outcomes

Decision tree–based classifiers provide insights on different participant groups who show promising results following ICBT. After identifying the two most optimal models, the model-agnostic posthoc framework Shapley additive explanations (SHAP) was used for analyzing ICBT outcome predictors [46,47]. This framework facilitates model interpretations and identifies the most influential factors that result in successful ICBT outcomes (ie, a reduction in TFI scores following the ICBT intervention). SHAP measure the impact of variables and take into account variables’ interactions with other variables. SHAP values indicate the importance of a feature and are calculated by comparing model predictions that account and do not account for a given feature. However, since the order in which a model sees features can affect its predictions, this comparison is done in every possible order, so that the features are compared in a fair manner.

##### Step 4: Identification of Participants Who Are Most Likely to Benefit From ICBT

The CART decision tree model was used to identify the participants who were the most (or least) likely to benefit from ICBT. In training, a minimum split of 20 and a max depth of 10 were used as the control parameters for the CART decision tree models. Tree pruning was conducted to reduce the overfitting in the CART decision tree models, although the best decision tree model remains the same even after pruning.

The data analysis was performed with R version 4.0.3 (R Foundation for Statistical Computing) software. The code is available in the GitHub repository for this study [48]. The data can be made available upon reasonable request.

## Results

### ICBT Effects

Undertaking ICBT significantly reduced tinnitus severity scores (t_227_=16.37; *P*<.001) from a mean baseline severity score of 57.93 (SD 19.17) to a mean post-ICBT severity score of 34.22 (SD 22.78), as measured by the TFI. A clinically significant 13-point change in TFI scores was achieved by 150 of the 228 participants (65.8%) after the intervention.

### Decision Tree Model Performance Evaluations

Table 1 contains the model evaluation information of all 6 decision tree classifiers that were based on the test data. Following training via the 3-fold cross-validation method, the mean accuracies of the six decision tree classifiers ranged between a minimum of 56.3% (the C5.0 model) to a maximum of 71.8% (the GB model). Model predictions showed variations in their sensitivity (range 68.6%-78.3%), specificity (range 31.1%-64%), and AUC values (range 0.52-0.69).

**Table 1:** Decision tree model evaluations.

Classification model	Accuracy (%), mean (SD)	Sensitivity (%; true positive rate), mean (SD)	Specificity (%; true negative rate), mean (SD)	AUC^a^, mean (SD)
Classification and regression decision tree	70.7 (2.4)	74 (5.5)	64 (3.7)	0.69 (0.001)
C5.0	56.3 (1.1)	68.6 (1.9)	31.1 (6.3)	0.52 (0.001)
Gradient boosting	71.8 (1.5)	78.3 (2.8)	58.7 (4.2)	0.68 (0.02)
Extreme gradient boosting	65 (4.1)	77.9 (8.7)	39.2 (6.6)	0.62 (0.08)
AdaBoost algorithm	63.6 (3.2)	73.3 (5.2)	44 (7.8)	0.58 (0.05)
Random forest	66.7 (3)	75 (6.1)	50 (7.2)	0.60 (0.01)

^a^AUC: area under the receiver operating characteristic curve.

None of the six models were considered robust, as their AUC values were below 0.80. As the CART and GB classifiers were found to be superior compared to the other four models when considering all evaluation measurements as a whole (accuracy, sensitivity, specificity, and the AUC), these two models were further examined.

### Feature Importance

**Figure 1:**
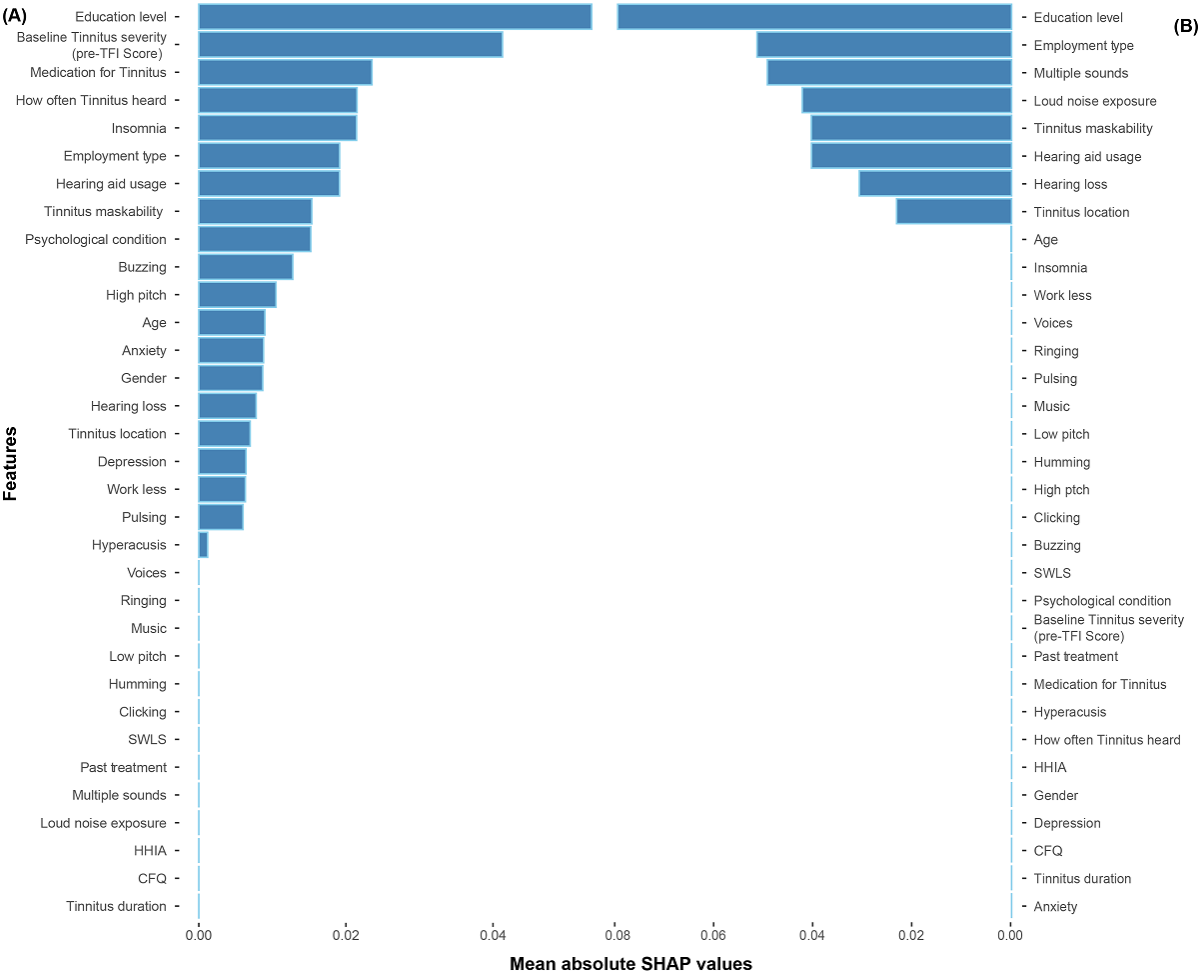
Feature importance based on the mean absolute SHAP values from (A) the best gradient model and (B) from the best classification and regression tree decision tree model. These SHAP values represent the absolute change in log odds. Relatively higher importance is indicated with larger SHAP values. CFQ: Cognitive Failures Questionnaire; HHIA: Hearing Handicap Inventory for Adults; SHAP: Shapley additive explanations; SWLS: Satisfaction with Life Scales; TFI: Tinnitus Functional Index.

The SHAP framework was applied to the CART and GB classifiers to estimate each predictor variable's importance in predicting ICBT outcomes (Figure 1). Variables with larger SHAP values are relatively more important in terms of their contributions (ie, feature contribution) to a model prediction. Education level (average SHAP values: GB model=0.053; CART model=0.079) was identified as the most important influencing factor in both models. Although they were not ranked in the same order, the other features that ranked within the top 10 features for both models were employment type (average SHAP values: GB model=0.019; CART model=0.051), hearing aid usage (average SHAP values: GB model=0.019; CART model=0.040), and tinnitus maskability (average SHAP value: GB model=0.015; CART model=0.040). The differences between these models were that the GB model ranked baseline tinnitus severity (average SHAP value=0.041), how often tinnitus is heard (average SHAP value=0.022), insomnia (average SHAP value=0.021), the use of medication for tinnitus (average SHAP value=0.024), and the presence of a psychological condition (average SHAP value=0.015) among the top 10 features, whereas the CART model ranked the presence of multiple sounds (average SHAP value=0.049), loud noise exposure (average SHAP value=0.042), and tinnitus location (average SHAP value=0.023) as key features.

**Figure 2:**
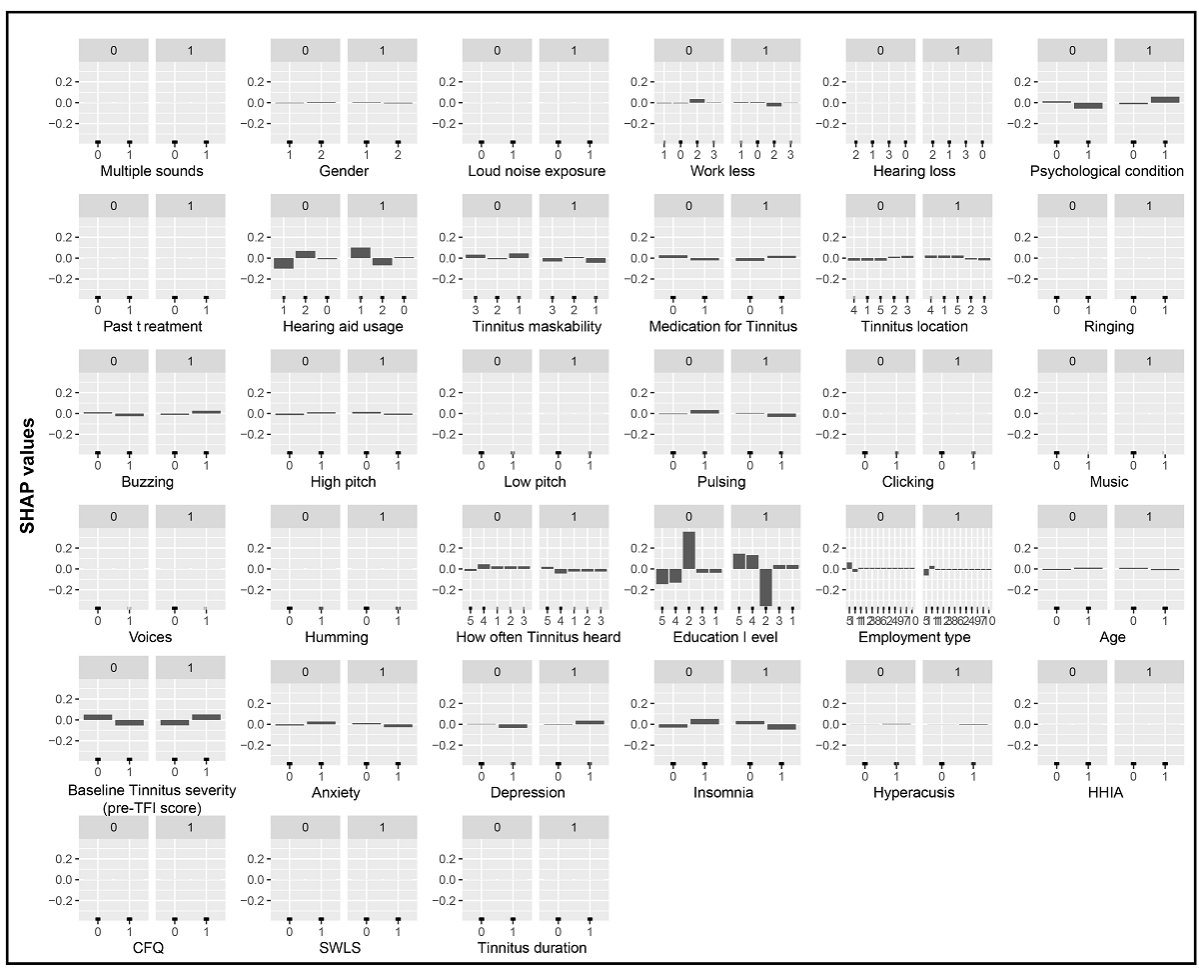
The best gradient boosting model–based feature effects. Each graph represents a feature and their corresponding SHAP values. Plots labeled with “1” illustrate the impact that each feature has on achieving a successful treatment outcome (a 13-point or more reduction in TFI score). CFQ: Cognitive Failures Questionnaire; HHIA: Hearing Handicap Inventory for Adults; SHAP: Shapley additive explanations; SWLS: Satisfaction with Life Scales; TFI: Tinnitus Functional Index.

**Figure 3:**
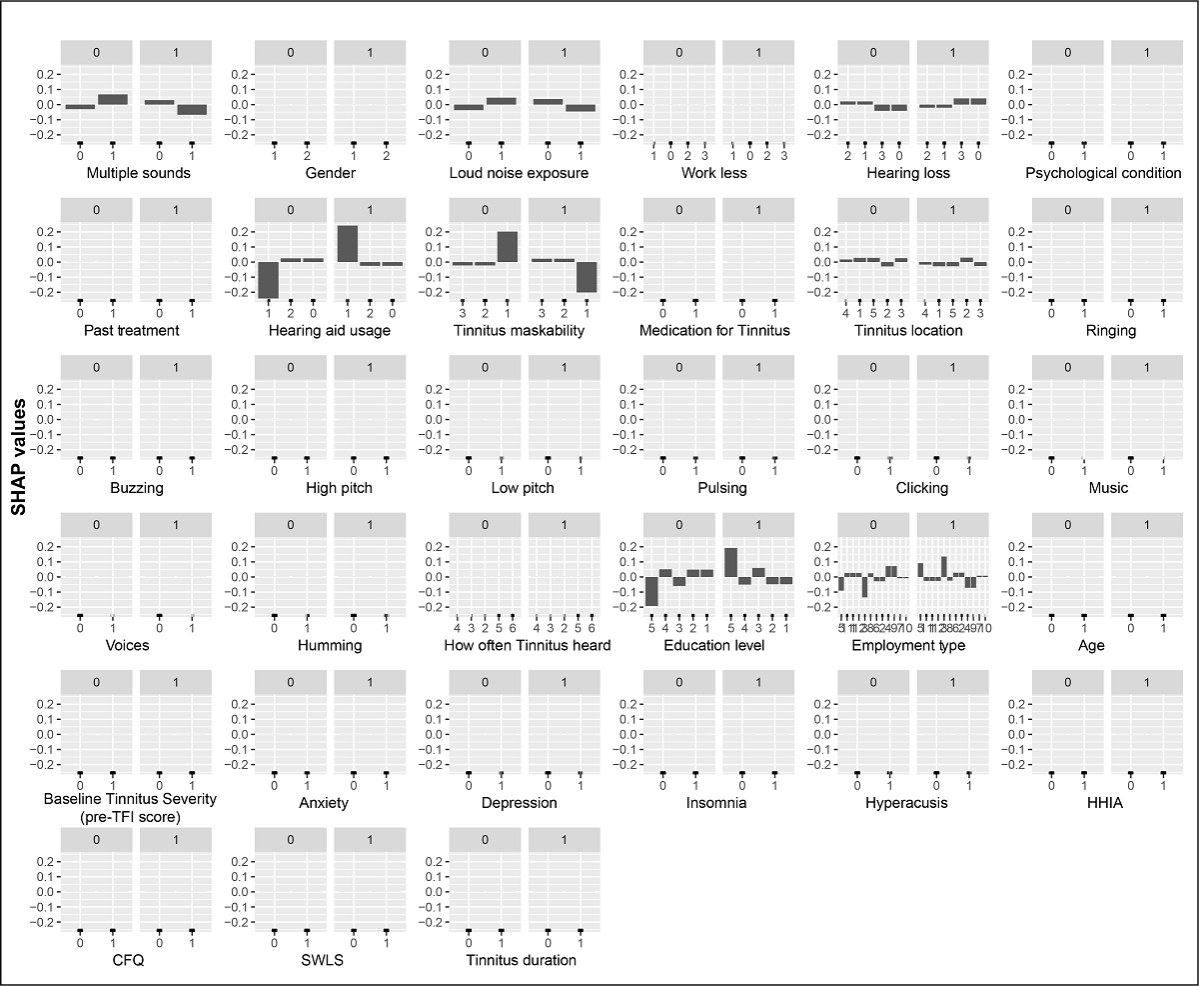
The best classification and regression tree decision tree model feature effects. Each graph represents a feature vs. corresponding SHAP value. Plots with “1” represent the effect that each feature has on achieving a successful treatment outcome. CFQ: Cognitive Failures Questionnaire; HHIA: Hearing Handicap Inventory for Adults; SHAP: Shapley additive explanations; SWLS: Satisfaction with Life Scales; TFI: Tinnitus Functional Index.

Figures 2 and 3 present the effect that each feature category had on the outcome variable, as decided by the best GB and CART decision tree models. Features’ impacts on the two classes are presented in 2 separate plots for each feature (“1” indicates the effect on the successful treatment class and “0” indicates the effect on the unsuccessful treatment class). Positive SHAP values in each successful treatment group indicate a higher log odd of achieving a 13-point or more tinnitus severity score reduction (ie, on the TFI for a given category) for a feature and vice versa. This log odd is relative to the training set average. Figures 2 and 3 both depict positive SHAP values for the participants who had a vocational training degree or a master’s degree (or above), those with higher levels of education (postgraduate degrees), and those who were using a hearing aid in only 1 ear. As per the GB model, this reduction was more likely to occur for participants with insomnia (scores of 14 or less on the Insomnia Severity Index), a psychological condition, and tinnitus that can be described as a buzzing sound and for participants who had median baseline tinnitus severity scores (ie, pre-TFI assessment) of >55.2.

### Identification of Participants Who Are Likely to Benefit From ICBT

**Figure 4:**
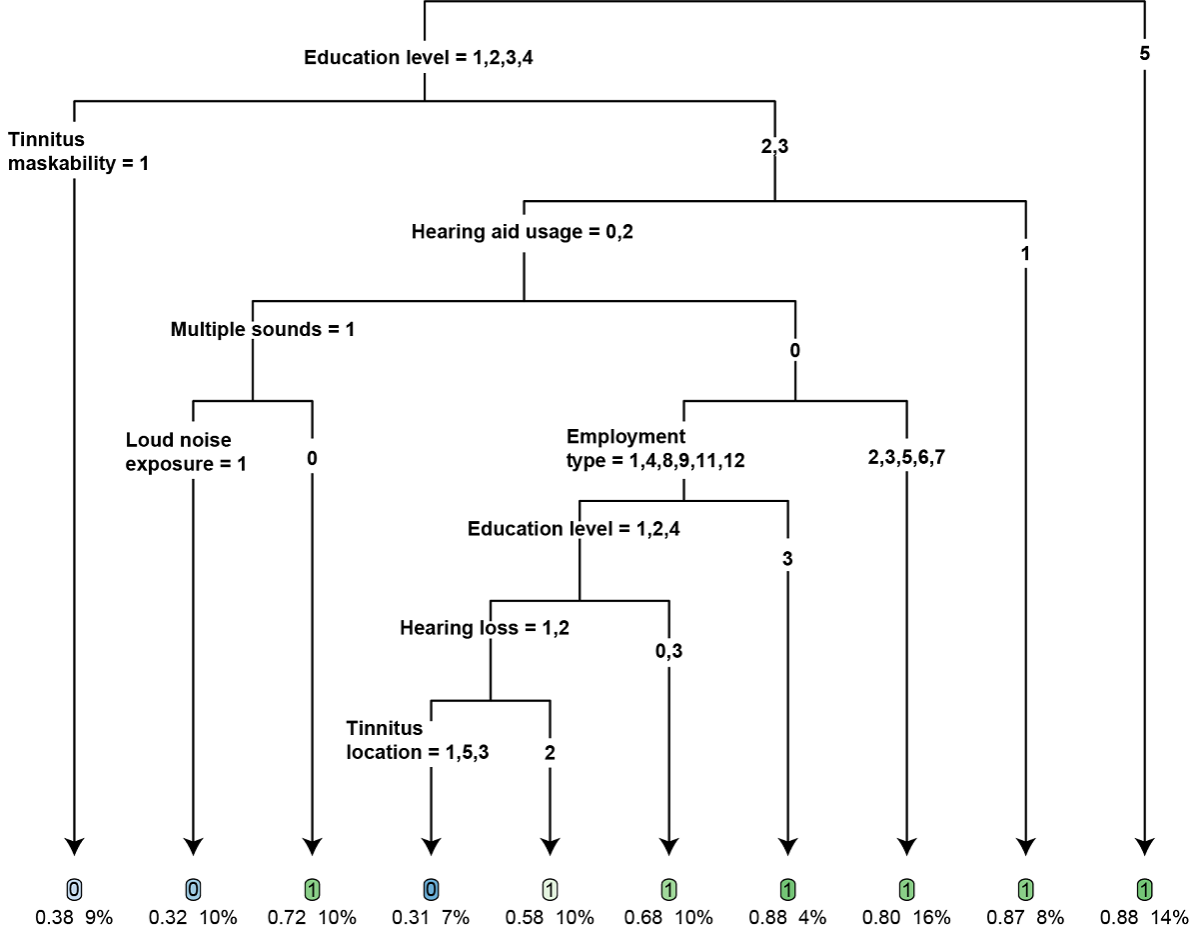
The best classification and regression tree decision tree model. The fitted tree has 10 terminal nodes (denote the decision criteria). The variable categories (please refer to Multimedia Appendix 2 for variable category labels) corresponding to each split are given at the top of each branch. Each terminal node contains the predicted treatment class ("1" indicates the successful treatment class and "0" indicates the unsuccessful treatment class), the percentage of subjects with successful treatment outcomes, and the percentage of participants with the given characteristics in the training set.

Figure 4 presents the final decision tree model, which has 10 nodes. A detailed explanation is provided in Multimedia Appendix 3. In this model, homogenous groups were formed by creating binary splits at each node. The decision nodes represent the treatment groups (either 0 or 1) that were the most likely to achieve tinnitus severity reduction, and the decisions in the tree were based on the characteristics of each group. These characteristics were represented by each branch (based on their corresponding feature values) of the tree. This model showed that higher education level, tinnitus maskability, hearing aid usage, the presence of multiple tinnitus sounds, loud noise exposure, employment type, the presence of hearing loss, and tinnitus location were important factors for determining treatment outcomes. The following participant groups had at least an 85% chance of achieving a TFI score reduction of 13 points or more:

- Participants with postgraduate education (master’s degree or higher)
- Participants with an education level other than a master’s level of education and poor tinnitus maskability (or only partial tinnitus maskability) and those who wore a hearing aid in 1 ear
- Participants with no tinnitus maskability or only partial tinnitus maskability; those who did not wear a hearing aid or used hearing aids bilaterally; those who did not hear multiple tinnitus sounds; and those with an occupation that could described as *professional*, *technical*, *skills based* (ie, skilled tradesman), *service related*, or *medical*

## Discussion

The aim of this study was to explore predictors of outcomes in ICBT for tinnitus by applying 6 types of decision tree models to a combined data set of participants from 3 clinical trials. The key findings are discussed below.

### Best Decision Tree Models

In this study, we applied 6 different decision tree models to ICBT data. Although none of the six models attained an excellent or outstanding status, the CART and GB models’ discriminative power can be considered to be satisfactory, given the moderate sample size with just 33 predictive factors (features). This is consistent with a recent study that used 10 decision tree models to predict the outcomes of CBT for patients with tinnitus (N=1416) and found that the GB model with 26 predictive factors had the best predictive power; it had an AUC value of 0.89 [21].

Further work however is needed in this area to determine which models and how many vital factors may result in an optimal predictive model. A larger sample size would likely improve the results. However, we are not sure if just adding factors would be helpful. This is because Niemann et al [21] included 205 factors in their analysis, and of these, only 26 were helpful in achieving optimal results. Moreover, of the 26 factors, only a handful had the most considerable effect. For instance, only 1 factor (ie, tinnitus impairment in terms of loudness, frequency, and distress) resulted in an AUC of 0.79, only 3 features resulted in an AUC of 0.85, and only 8 factors resulted in an AUC of 0.85. These results indicate that including key factors with high predictive power may be a better approach than just adding all of the possible factors.

### Predictors of ICBT Outcomes

Among the best decision tree models (ie, the CART and GB models), various factors were found to be critical predictors of ICBT outcomes. These included demographics (ie, education level, employment type, and the presence of a psychological condition), tinnitus and hearing-related factors (ie, baseline tinnitus severity, tinnitus location, how often tinnitus is heard, a buzzing type of tinnitus, tinnitus maskability, and hearing loss type), treatment-related factors (ie, hearing aid usage), and clinical factors (ie, insomnia). However, education level was the most notable predictor among these.

Participants who had a master’s degree or above had an 88% chance of achieving a successful outcome. This is understandable, as the ability to read, understand, and follow instructions is key for undergoing self-help interventions. However, it is likely that the way in which the materials were written may also have played a role. For instance, UK ICBT materials are written at a ninth-grade reading level [49], which may require higher literacy skills. However, these materials have been rewritten at a sixth-grade reading level and below [49] to ensure accessibility for those with lower education levels. More and more people, including those with lower education, are using the internet and participating in internet-based treatments, particularly due to the constraints that have been placed on health care during the COVID-19 pandemic [50-52]. In our sample, over 85% (198/228, 86.8%) of the participants had a below–master-level education. This highlights the need for making ICBT more accessible to increase the chances of achieving improved outcomes for those with lower education levels.

Baseline tinnitus severity was found to be another critical factor for predicting ICBT outcomes in the GB model. Our previous studies on 1-year outcomes [53] and our previous application of univariate and multivariate analyses to this study’s sample [19] identified baseline tinnitus severity as a critical predictive variable. In the Niemann et al [21] study, tinnitus loudness, frequency, and distress, which were measured by using a visual analog scale, were found to be key predictive factors. Further, tinnitus distress, which was measured by using the German version of the Tinnitus Questionnaire (a tool that is comparable to the TFI in this study), was not found to be the key predictive factor. However, based on both clinical experiences and findings from many previous studies, baseline tinnitus severity is an important factor for determining treatment outcomes. The clinical factors depression and anxiety were among the key predictive factors in the GB model. A recent clinical trial conducted by Beukes et al [54], as well as the Niemann et al [21] study, determined that those with high levels of depression had a better chance of achieving success.

Although various other tinnitus and hearing-related variables could have played a role in determining the outcomes of ICBT, the predictive power of our models was relatively low based on the AUC values. Nevertheless, it would be useful for hearing health care professionals to examine these factors when deciding on the candidacy of self-help psychological interventions such as ICBT. Moreover, it would be useful for future studies to examine any additional factors (eg, health literacy) that may have a bearing on ICBT outcomes.

### Study Limitations and Future Directions

Although this study is among the first to apply data mining models to ICBT data, it has several limitations. The sample size was limited, and this may have contributed to the low predictive accuracies of the models. The exploratory decision tree models worked better when including a large number of predictive factors. In this study, we only included 33 predictive factors in our models, and this may have limited the performance of our models. Further, we may have missed some important factors (eg, health literacy) that have a bearing on ICBT outcomes.

The inclusion and exclusion criteria that were used in the three trials from which our data were generated may have resulted in a sample with high tinnitus severity levels that may not be representative of the general tinnitus population. This may have also contributed to our limited key findings. Future studies could include more extensive samples of heterogeneous patients with tinnitus as well as all of the possible predictive factors that could help with improving our models’ predictive power. Moreover, developing nonlinear classifiers with artificial neural networks and support vector machines could help with achieving higher prediction accuracies and should be examined in future studies.

In conclusion, tree models, especially the CART and GB models, appear to be promising in predicting ICBT outcomes. Future studies should be undertaken with larger sample sizes and include a more comprehensive range of predictive factors to improve their models’ predictive power.

## Appendix

Multimedia Appendix 1 Characteristics of the study's participants.

Multimedia Appendix 2 Predictor variables.

Multimedia Appendix 3 Detailed explanation of classification and regression tree model.

## Abbreviations

AUC: area under the receiver operating characteristic curve
CART: classification and regression tree
CBT: cognitive behavioral therapy
GB: gradient boosting
ICBT: internet-based cognitive behavioral therapy
RF: random forest
SHAP: Shapley additive explanations
TFI: Tinnitus Functional Index
XGBoost: extreme gradient boosting
