# Supplementary material for: Exploratory Data Mining Techniques (Decision Tree Models) for Examining the Impact of Internet-Based Cognitive Behavioral Therapy for Tinnitus: Machine Learning Approach
Source: J Med Internet Res. 2021 Nov 2;23(11):e28999. doi: 10.2196/28999 (PMC8596228; doi:10.2196/28999)
Supplement: Multimedia Appendix 1 [file jmir_v23i11e28999_app1.docx]

**Appendix 1: Participant’s characteristics**

Table 1.1: Characteristics of the study's participants

| **Characteristic** | **N (%)** | **Mean (SD)** |
| --- | --- | --- |
| **Demographic characteristics** | | |
| Age (in years) |  | 55.14 (12.92) |
| Gender   - Female - Male | 98 (43%)  130 (57%) |  |
| Highest level of education   - High school or below - College - Vocational training - Bachelor’s degree - Masters degree or above | 59 (25.9%)  47 (20.6%)  31 (13.6%)  61 (26.8%)  30 (13.2%) |  |
| Employment   - Manager - Professional - Technical - Administrative - Skilled tradesman - Service occupation - Medical - Sales - Homemaker - Student - Retired - Unemployed | 27 (11.8%)  46 (20.2%)  16 (6%)  17 (7%)  11 (4.8%)  11 (4.8%)  6 (2.6%)  8 (3.5%)  4 (1.8%)  1 (0%)  73 (32%)  11 (4.8%) |  |
| Loud noise exposure   - Yes - No | 103 (45.2%)  125 (54.8%) |  |
| Diagnosed with a psychological condition   - Yes - No | 50 (21.9%)  178 (78.1%) |  |
| Working less due to tinnitus   - Reduced hours - Stopped work - Disability allowance - No | 8 (3.5%)  32 (14%)  7 (3.1%)  181 (79.4%) |  |
| **Tinnitus and hearing-related characteristics** | | |
| Baseline tinnitus severity (aka: Pre-TFI, measured using Tinnitus Functional Index) |  | 57.93 (19.17) |
| Tinnitus duration (in years) |  | 17.68 (19.42) |
| How often tinnitus is heard   - Occasionally - When taking out my hearing aid(s) - At night - Most of the time - All the time | 4 (1.8%)  3 (1.3%)  4 (1.8%)  63 (27.6%)  154 (67.5%) |  |
| Tinnitus location   - One ear - Both ears - In my head - Other location - Unsure | 61 (26.8%)  109 (47.8%)  34 (14.9%)  3 (1.3%)  21 (9.2%) |  |
| Type of tinnitus sound (answering Yes)   - Ringing - Buzzing - High pitched sound - Low pitched sound - Pulsating - Clicking - Music - Voices - Humming | 71 (31.1%)  75 (32.9%)  130 (57%)  16 (7%)  28 (12.3%)  14 (6.1%)  4 (1.8%)  3 (1.3%)  21 (9.2%) |  |
| Multiple sounds heard   - Yes - No | 73 (32%)  155 (68%) |  |
| Presence of a hearing loss   - No - Both ears - One ear - Unsure | 49 (21.5%)  104 (45.6%)  46 (20.2%)  29 (12.7%) |  |
| **Treatment-related characteristics** | | |
| Past tinnitus treatment sought   - Yes - No | 58 (25.4%)  170 (74.6%) |  |
| Sounds can distract from tinnitus (tinnitus maskability)   - Fully - Partially - Not at all | 26 (11.4%)  178 (78.1%)  24 (10.5%) |  |
| Hearing aid use   - No - Unilateral - Bilateral | 159 (69.7%)  19 (8.3%)  50 (21.9%) |  |
| Medication use   - Yes - No | 130 (57%)  98 (43%) |  |
