# Supplementary material for: Exploratory Data Mining Techniques (Decision Tree Models) for Examining the Impact of Internet-Based Cognitive Behavioral Therapy for Tinnitus: Machine Learning Approach
Source: J Med Internet Res. 2021 Nov 2;23(11):e28999. doi: 10.2196/28999 (PMC8596228; doi:10.2196/28999)
Supplement: Multimedia Appendix 2 [file jmir_v23i11e28999_app2.docx]

**Appendix 2: Predictor Variables**

Table 2.1: Demographic variables (7 variables)

| **Variable** | **Question** | **Response options** |
| --- | --- | --- |
| Age | What is your age? | In years  Split into dichotomous variables (<=57 years of age and >57 years of age) based on the median |
| Gender | What is your gender? | Male (1), Female (2) |
| Education level | What is the highest level of education you have completed? | Highschool or less (1), College (2), Vocational training (3), Bachelor’s degree (4), Master’s degree or above (5) |
| Employment type | What best describes your employment? | Manager (1), Professional (2), Technical (3), Administrative (4), Skilled tradesman (5), Service occupation (6), Medical (7), Sales (8), Home maker (9), Student (10), Retired (11), Unemployed (12) |
| Loud noise exposure | Have you been exposed to loud noise? | Yes (1) , No (0) |
| Diagnosed with psychological condition | Have you been presently diagnosed with any psychological conditions including anxiety and depression? | Yes (1) , No (0) |
| Work less due to tinnitus | Do you work less because of your tinnitus? | No (0), Reduced hours (1), Stopped work (2), Disability allowance (3) |

Table 2.2: Tinnitus and hearing-related variables (15 variables)

| **Variable** | **Question** | **Response options** |
| --- | --- | --- |
| Baseline tinnitus severity (Pre-TFI) | Measured using the Tinnitus Functional Index (TFI) | Scores range from 0 to 100.  Split into dichotomous variables (<=55.2 and >55.2) based on the median |
| Tinnitus duration | How long have you had tinnitus for? | In years  Split into dichotomous variables (<=10.00 years and >10.00 years) based on the median |
| How often is tinnitus heard? | How often is tinnitus heard? | Occasionally (1), When taking out my hearing aid(s) (2), At night (3), Most of the time (4), All the time (5) |
| Tinnitus location | Where do you notice your tinnitus? | One ear (1), Both ears (2), In my head (3), Unsure (4), Other (5) |
| Type of tinnitus (9 different types) | - Ringing - Buzzing - High pitched sound - Low pitched sound - Pulsing - Clicking - Music - Voices - Humming | For each item: Yes (1) , No (0) |
| Multiple tones heard | This variable is computed based on responses to types of tinnitus. Answer yes to multiple types of tinnitus was considered as multiple tones heard | Yes (1) , No (0) |
| Presence of a hearing loss | Do you have a hearing loss? | No (0), Both ears (1), One ear (2), Unsure (3) |

Table 2.3: Treatment-related variables (4 variables)

| **Variable** | **Question** | **Response options** |
| --- | --- | --- |
| Past treatment sought | Have you received treatment for tinnitus in the past? | Yes (1) , No (0) |
| Sounds can distract from tinnitus (tinnitus maskability) | How well can sounds around you distract you from your tinnitus or make the tinnitus less noticeable? | Fully (1), Partially (2), Not at all (3) |
| Hearing aid use | Do you wear hearing aid(s) or any other amplification devices? | No (0), One ear (1), Both ears (2) |
| Medication use | Do you currently take any medications? | Yes (1) , No (0) |

Table 2.4: Clinical factors (7 variables)

| **Variable** | **Questionnaire** | **Number of items/ Response options** | **Score** |
| --- | --- | --- | --- |
| Anxiety | General Anxiety Disorders (GAD-7) | 7-items  4-point scale with “not at all” (score of 0) to “nearly every day” (score of 3) | Higher number indicates more severe anxiety (scores range between 0–21). The total score is interpreted as follows:   - 0–4: minimal anxiety - 5–9: mild anxiety - 10–14: moderate anxiety - 15–21: severe anxiety   Split into dichotomous variables (<=9 no anxiety and >9 anxiety) |
| Depression | Patient Health Questionnaire (PHQ-9) | 9-items  4-point scale with “not at all” (score of 0) to “nearly every day” (score of 3) | Higher number indicates more severe depression (scores range between 0–27).  The total score is interpreted as follows:   - 5–9: mild depression - 10–14: moderate - 15–19: moderately severe - 20–18: severe depression   Split into dichotomous variables (<=14 no depression and >14 depression) |
| Insomnia | Insomnia Severity Index (ISA) | 7-item  5-point scale with “no problem” (score of 0) to “very severe problem” (score of 4) | Higher number indicates more severe insomnia (scores range between 0–28).  The total score is interpreted as follows:   - 0–7: not clinically significant - 8–14: subthreshold insomnia - 15–21: clinical insomnia (moderate severity) - 22–28: clinical insomnia (severe degree)   Split into dichotomous variables (<=14 no insomnia and >15 insomnia) |
| Hyperacusis | Hyperacusis Questionnaire (HQ) | 14-items  4-point scale with “no” (score of 0) to “yes, a lot” (score of 3) | Higher number more severe hyperacusis (scores range between 0–42).  The total score is interpreted as follows:   - >28: strong hypersensitivity   Split into dichotomous variables (<=28 no hyperacusis and >28 hyperacusis) |
| Hearing disability | Hearing Handicap Inventory for Adults – Screening (HHIA-S) | 10-items  3-point scale with “yes” (score of 4) to “no” day (0) | Higher number more severe hearing disability (scores range between 0–40).  The total score is interpreted as follows:   - 0–8: no hearing disability - 10–24: mild to moderate hearing disability - 26–40: severe hearing disability   Split into dichotomous variables (<=8 no hearing disability and >=10 hearing disability) |
| Cognitive failures | Cognitive Failures Questionnaire (CFQ) | 25-items  5-point scale with “never” (score of 0) to “very often” (score of 4) | Higher scores indicate more difficulties (cognitive failures) in perception, memory, and motor function (score range 0–100).  The total score is interpreted as follows:  The scores range 0–100 with higher scores indicating more cognitive failures/problems (or reduced cognitive functioning).  Split into dichotomous variables (<=32 no cognitive problems and >32 cognitive problems) |
| Life satisfaction | Satisfaction with Life Scale (SWLS) | 5-items  7-point scale with “strongly disagree” (score of 1) to “strongly agree” (7) | Higher number indicated more satisfaction with life (scores range between 5–35).  The total score is interpreted as follows:   - 0–9: extremely dissatisfied - 10–14: dissatisfied - 15–19: below average satisfaction - 20–24: average satisfaction - 25–29: high satisfaction - 30–35: highly satisfied   Split into dichotomous variables (<=19 life satisfaction and >19 high satisfaction) |
