# Supplementary material for: Exploratory Data Mining Techniques (Decision Tree Models) for Examining the Impact of Internet-Based Cognitive Behavioral Therapy for Tinnitus: Machine Learning Approach
Source: J Med Internet Res. 2021 Nov 2;23(11):e28999. doi: 10.2196/28999 (PMC8596228; doi:10.2196/28999)
Supplement: Multimedia Appendix 3 [file jmir_v23i11e28999_app3.docx]

**Appendix 3: Detailed explanation of the best CART decision tree model**

Figure 4 in the manuscript presents the final CART decision tree model which showed that education level, sounds can distract, hearing aid usage, multi-sounds, loud noise exposure, employment type, hearing loss, and tinnitus location to be important factors. In fact, SHAP analysis discussed above had identified these factors to be the most influential features contributing to the model outcome. In the following, we summarize the characteristics of the participant groups who were most likely to be benefitted from the ICBT intervention.

- If a participant has an education of master’s degree or above, he/she has approximately 88% chance of having successful outcome with the ICBT intervention. 14% of the study participants were in this category.
- If a participant has a different education level other than master’s, and if she/he is not distracted at all or has a partial distraction by the sounds from the tinnitus, and if he/she uses hearing aid in his/her single ear, he/she has about 87% chance of success. 8% of the study participants were in this category.
- If a participant has a different education level other than master’s, and if she/he is not distracted at all or has a partial distraction by the sounds from the tinnitus, and if he/she uses no hearing aid or hearing aids for both ears, but no multiple tones were heard and works in one of the following; professional, technical, skilled tradesman, service occupation or medical, then he/she has about 80% chance of success. 16% of the study participants were in this category.
- If a participant is not distracted at all or has a partial distraction by the sounds from the tinnitus, and if he/she uses no hearing aid or hearing aids for both ears, but no multiple tones were heard and works in one of the following; manager, administrative, sales, home maker, student, retired or unemployed whom had a vocational training, then he/she has about 88% chance of success. Only 4% of the study participants were in this category.
- If a participant is not distracted at all or has a partial distraction by the sounds from the tinnitus, and if he/she uses no hearing aid or hearing aids for both ears, but no multiple tones were heard and works in one of the following; manager, administrative, sales, home maker, student, retired or unemployed whom had an education either high school or less, college or bachelor’s degree, and he/she has no hearing loss or if it is unsure, then he/she has about 68% chance of success. 10% of the study participants were in this category.
- If a participant is not distracted at all or has a partial distraction by the sounds from the tinnitus, and if he/she uses no hearing aid or hearing aids for both ears, but no multiple tones were heard and works in one of the following; manager, administrative, sales, home maker, student, retired or unemployed whom had an education either high school or less, college or bachelor’s degree, and he/she has a hearing loss either in one ear or both, and he/she recognized his/her tinnitus location to be in their head, then he/she has about 58% chance of success. 10% of the study in this category.
- If a participant has a different education level other than master’s and he/she is not distracted at all or has a partial distraction by the sounds from the tinnitus, and if he/she uses no hearing aid or hearing aids for both ears, and he/she hears multiple tones and have not had a loud noise exposure, then he/she has about 72% chance of success. 10% of the study participants were in this category.

Next, we summarize the characteristics of the subject groups whom least likely to be benefitted with the ICBT treatment.

- If a participant has a different education level other than masters’, and if she/he is fully distracted by the sounds, then he/she has about 38% chance of success. 9% of the study participants were in this category.
- If a subject has a different education level other than master’s and he/she is not distracted at all or has a partial distraction by the sounds from the tinnitus, and if he/she uses no hearing aid or hearing aids for both ears, and he/she hears multiple tones and had a loud noise exposure, then he/she has about, only 32% chance of success. 10% of the study participants were in this category.
- If a subject is not distracted at all or has a partial distraction by the sounds from the tinnitus, and if he/she uses no hearing aid or hearing aids for both ears, but no multiple tones were heard and works in one of the following; manager, administrative, sales, home maker, student, retired or unemployed whom had an education either high school or less, college or Bachelor’s degree, and he/she has a hearing loss either in one ear or both, and he/she recognized his/her tinnitus location to be in somewhere other than the head, then he/she has about 31% chance of success. Only 7% of the study participants were in this category.

These subject groupings had shown consistent probabilities in majority of the CART decision tree models that were generated with different random initializations.
